# Extramedullary Plasmacytoma of the Frontal Sinus: Case Report and Turkish Literature Review

**DOI:** 10.4274/tjh.2012.0175

**Published:** 2014-09-05

**Authors:** Ayşegül Verim, Shahrouz Sheidaii, Ömer Bilaç, Çiğdem Tepe Karaca, Barış Naiboğlu

**Affiliations:** 1 Haydarpaşa Numune Training and Research Hospital, Clinic of Otorhinolaryngology, İstanbul, Turkey

**Keywords:** Extramedullary plasmacytoma, Frontal sinus, Mucocel, Sinonasal neoplasm

## Abstract

Solitary extramedullary plasmacytomas (EMPs) are nonepithelial neoplasms of plasma cell origin categorized among non-Hodgkin lymphomas, without the bone marrow involvement and systemic spread seen in multiple myeloma. They are uncommon tumors comprising 3% of all plasma cell neoplasias. Although they usually occur in the upper respiratory tract, only 1 case of EMP localized to the frontal sinus has been reported in the English literature. We present in this report a rare case of EMP originated from the left frontal sinus leading to left eyeball proptosis and movement restriction. A survey of sinonasal EMPs in the Turkish literature is reported, as well. Paranasal computerized tomography and magnetic resonance imaging of a 69-year-old female who presented with left eyeball proptosis and left-sided headache revealed a solid mass in the left frontal sinus. Histopathological analysis of the completely excised mass supported the diagnosis of plasmacytoma. The definitive diagnosis of solitary EMP was confirmed with further investigations at hematology and oncology clinics. The patient was treated with surgery followed by local radiotherapy to the head and neck region, and she was disease-free at her 1-year follow-up. Treatment of sinonasal EMP is surgery alone or surgery combined with radiotherapy. Long-term follow-up is a requisite for systemic control because of the disease’s high potential to transform into multiple myeloma.

## OZET

Ekstrameduller soliter plazmasitomlar plazma hücrelerinden kaynaklanan, non Hodgkin lemfomalar arasında sınıflandırılmış, kemik iliğini tutmayan ve multipl miyelom gibi jeneralize olmamış formda non epitelyal tümörlerdir. Nadir görülen tümörler olup plasma hücreli neoplazilerin %3’ünü oluştururlar. Çoğunlukla üst solunum yolunda sinonazal yerleşimli olmasına rağmen frontal sinüs yerleşimli olanına literatürde 1 olguda rastlanılmıştır. Bu yazıda sol frontal sinüs yerleşimli sol gözde proptoz ve hareket kısıtlılığına sebep olan nadir bir extrameduller soliter plazmasitom olgusu sunulmakta ve Türk literatüründeki sinonazal yerleşimli EMP’ler gözden geçirilmektedir. Altmış dokuz yaşında baş ağrısı ve sol gözde dışa itilme yakınması ile gelen bayan hastada çekilen paranazal BT ve MR sol frontal sinüs kitlesini ortaya koymuştur. Açık cerrahi ile tamamı çıkarılan kitlenin histopatolojik analizi plazmasitom tanısını koydurmuştur. Soliter EMP tanısı hematoloji ve onkoloji klinikleri tarafından yapılan tetkiklerle kesinleşmiştir. Hastaya postoperatif radyoterapi uygulanarak olumlu yanıt elde edilmiştir. Hasta halen sağlıklı olup 18. ay kontrolunda nüks izlenmemiştir. Sinonazal yerleşimli ekstramedüller plazmasitomların tedavileri cerrahi, cerrahi ve/veya radyoterapidir. Uzun dönemde hastalığın multipl miyeloma dönüşme gibi bir potansiyeli olabileceği gözardı edilmeden takiplerinde tam bir sistemik kontrol yapılması zorunludur.

## INTRODUCTION

Plasmacytoma is a neoplasm consisting of proliferated monoclonal plasma cells in either the bone or soft tissue. It presents either as a single lesion (solitary plasmacytoma) or as multiple lesions localized in bone marrow [multiple myeloma (MM)]. Solitary plasmacytomas most frequently occur in the bone (solitary plasmacytoma of the bone), but they can also be found in the soft tissues as solitary extramedullary plasmacytomas (EMPs). 

EMPs are categorized as very rarely seen forms of the non-Hodgkin lymphomas. They represent about 3% of all plasma cell tumors [1]. The tonsils, nasal cavity, paranasal sinuses, nasopharynx, and trachea are the most commonly affected sites. Regional lymph node involvement occurs in 10% to 15% of cases [2].

The median age at diagnosis is 55 to 60 years, and approximately two-thirds of the patients are males [3].

A combination of surgery (for resectable tumors) and radiotherapy is the treatment of choice [4]. Chemotherapy is recommended for patients refractory to radiotherapy [5]. 

We present herein a 69-year-old woman who was referred to the ear, nose, and throat (ENT) clinic with a left-sided frontal mass and protrusion of the left eyeball due to an EMP originating from the left frontal sinus. A review of the Turkish literature on sinonasal plasmacytomas is also provided.

## CASE PRESENTATION

A 69-year-old woman was referred to the ENT clinic with left frontal swelling for 7 days. Her past medical history was unremarkable except for a chronic headache that had become refractory to analgesics in the last months and hypertension that was kept under control with a beta-blocker. No additional complaints such as epistaxis or nasal obstruction were presented.

On physical examination, a swelling in the left supraorbital region and forward protrusion of the left eyeball were noticed. The mass was smooth, nontender, and 4x3 cm in size. The anterior wall of the left frontal sinus was dehiscent. Visual acuity and eye movements were normal. Nasal endoscopy was unremarkable. No lymph node swelling was noticed on palpation.

**Figure 1:**
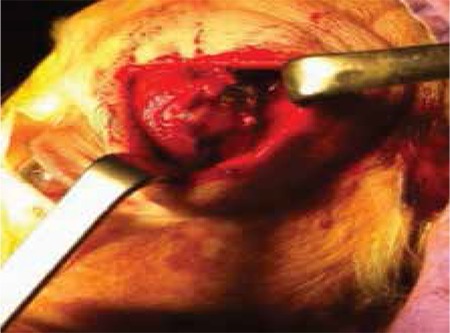
The mass seen through the dehiscent anterior wall of the left frontal sinus

Contrast-enhanced computerized tomography (CT) scanning demonstrated opacification of the left frontal sinus due to a soft tissue mass extending into the orbit (Figure 1). The anterior and inferior walls of the frontal sinus were dehiscent. The left eyeball was displaced anteroinferiorly.

**Figure 2:**
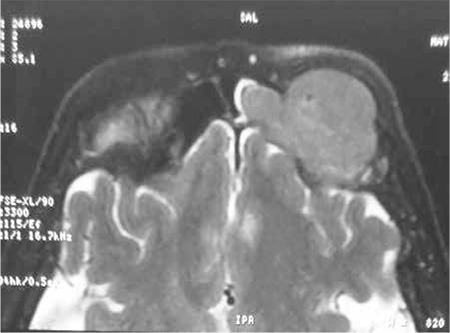
MRI images displaying the mass with extension into the orbit.

Magnetic resonance imaging (MRI) of the orbits revealed a large mass in the left frontal sinus extending into the orbit, isointense on T1A- and T2A-weighted sequences without involvement of the periorbital fat and extraocular muscles (Figure 2).

**Figure 3:**
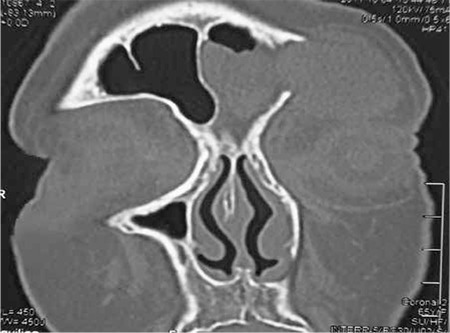
Images on paranasal sinus CT demonstrating the bony dehiscence.

The patient underwent left osteoplastic flap surgery. The lesion was nonencapsulated and multilobular (Figure 3). Frozen sectioning of the mass was consistent with plasmacytoma. The tumor was completely excised from the sinus cavity. Histopathological and immunohistochemical staining of the mass confirmed the diagnosis of plasmacytoma.

Postoperatively, the patient was referred to a hematology clinic for further diagnostic work-up, including bone marrow biopsy and aspiration, skeletal X-ray survey, serum electrophoresis, immunoglobulin quantification, serum and urine immunoelectrophoresis, β2-microglobulin assay, chest X-ray, and abdominal ultrasonography. The bone marrow biopsy showed a plasma cell infiltration of less than 5% of all nucleated cells. The absence of hypercalcemia, kidney failure, osteolytic bone lesions, and other organ involvement in a patient with localized disease suggested the diagnosis of solitary EMP. The blood work and urine electrophoresis results are given in Table 1.

The patient was given adjuvant radiotherapy at a dose of 40 Gy in the radiation oncology clinic. She was disease-free at her 18-month follow-up with no progression to MM.

## DISCUSSION AND REVIEW OF THE LITERATURE

EMP of the head and neck most commonly affects the sinonasal cavity’s mucosa (in more than 36% of all cases) [6]. The age range of patients is 17-80 years with a preponderance of males to females (2:1) [4].

The clinical presentation of paranasal sinus EMP depends on the mass volume and on the site of involvement. Nasal obstruction (80%), soft-tissue swelling, epistaxis, nasal discharge, pain, and proptosis are the main clinical findings observed in decreasing order of frequency. The tumor has a slow-growing nature and becomes symptomatic when the mass fills the cavity several months later. This condition results in treatment delay and transformation into systemic disease (MM). The early referral of the patient in this report was due to the obvious symptoms of the mass, which eroded the frontal sinus wall and pushed the eyeball anteroinferiorly. Had the sinonasal cavity been involved, the tumor would not have been diagnosed at an early stage and would have progressed into multiple myeloma, which usually has a fatal progression.

CT and MRI are complementary techniques in evaluating the size and location of a tumor and the involvement of the adjacent structures; however, the role of MRI in staging EMPs is not clear [7]. Although nonspecific, CT and MRI images show lobular soft tissue masses or infiltrative lesions with variable enhancement. Bony destruction is displayed depending on the expansion of the tumor [8]. Isointense images on T1-weighted and iso- to hyperintense images on T2-weighted sequences are well demonstrated in MRI assessment. In the current case, CT examination was useful in demonstrating the bony abnormality. MRI was superior to CT in defining the malignant character of the soft tissue mass, but it could not distinguish plasmacytoma from other probable causes (squamous cell or adenocystic carcinoma).

The diagnosis of EMP depends on the morphologic and immunophenotypic analysis of the localized tumoral mass [6]. As is the case in EMPs of the upper aerodigestive tract, sinonasal EMPs localize in the submucosal surface of the sinonasal cavity. Sinus mucosa may be thickened by inflammatory reactions; therefore, care should be taken to obtain deep biopsy specimens for frozen section analysis.

Immunophenotyping with CD15, CD20, CD45RB, CD45RA, epithelial membrane, IgA, IgD, IgG, IgM, IgE, kappa light chain, and lambda light chain antibodies is helpful to differentiate EMP from epithelial tumors [9]. Once the diagnosis was confirmed by histopathological analysis, our patient was referred to a hematology clinic for further diagnostic work-up, including serum and urine protein electrophoresis, immunoelectrophoresis, skeletal survey, and bone marrow biopsy, to look for systemic and bone marrow involvement.

Controversy exists regarding the optimal treatment of EMP. Based on the well-known high radiosensitivity of plasma cells, a dose of 40-50 Gy radiotherapy is advised in the treatment [10]. According to Alexiou et al., sinonasal EMPs with bone destruction should not be treated with radiotherapy or surgery alone, but rather with a combination of both modalities to assure local and systemic control [4]. Galieni et al. reported that surgery in cases of limited and easily resectable masses would be adequate to treat the disease without recurrence. They emphasized that the presence of a monoclonal component at diagnosis was a poor prognostic indicator for higher risk of disease progression [11].

Recently genetic abnormalities have become more apparent in pathogenesis and progression to MM. Additional biological factors related to the proliferative activity of the malignant plasma cells are high levels of angiogenesis and higher histologic grades. Translocations involving the immunoglobulin heavy-chain locus on chromosome 14q32, resulting in transcriptional activation of MAF, MMSET, CCND1, CCND3, or MAFB, and numerous structural and copy number alterations are important genetic changes for the pathogenesis of MM. Complex karyotypic abnormalities in MYC, the activation of NRAS and KRAS, mutations in TP53, and inactivation of CDKN2A and CDKN2C are late-onset translocations and gene mutations that promote a higher rate of progression to MM [12,13].

When compared to solitary bone plasmacytoma, EMP demonstrates a relatively low risk of progression to MM. The 10-year overall survival rate was reported to be 70% in EMP cases [14]. In another study, younger age was shown to be an independent good prognostic factor predicting a lower rate of progression to MM [15]. Tumor size of <5 cm, low M protein levels, patient age of <40 years, absence of light chains, and disappearance of M protein after treatment were found to be good prognostic factors [9,16]. Bone morphogenetic proteins (BMPs), members of the transforming growth factor-β superfamily and originally identified as molecules involved in the regulation of osteogenic differentiation, are now defined as tumor suppressors in actively proliferating myeloma cells or plasmablasts. Thus, activation of BMP6 expression is accepted to be a favorable prognostic factor independent of conventional prognostic factors, i.e. International MM Staging System stage and serum β2-microglobulin levels [17].

In the case of persistent M protein after completion of radiotherapy or suppression of normal immunoglobulin classes and suppression of BMP6 expression by intensive methylation of the genes at CpG sites of the BMP6 promoter region by plasma cells, with extremely high levels of serum lactate dehydrogenase, early intensive systemic therapy should be considered to prevent poor prognosis [18].

Because of the bony destruction in the present case, our treatment of choice was surgery combined with a radiotherapy dose of 40 Gy. The patient’s lesion was localized in only one sinus and was totally removed from the cavity. In our opinion, complete surgery with postoperative radiotherapy is more promising in the treatment of solitary EMP. The patient is now in follow-up at hematology and ENT clinics and is still free of disease at 18 months. Factors that influenced the good prognosis in our case were, as per the case-based experience in the literature, the relatively small size of the tumor (<5 cm), early presentation, a single site of involvement, localization (frontal sinus), female sex, and combined modality treatment.

Reports on nasal and paranasal sinus EMPs are rare in the English literature. Few reports have been published in the Turkish literature, as well. A comprehensive search of a Turkish database for patients with nasal and paranasal EMPs identified only 10 reports. Among these 10 reports, only 2 were included in PubMed [19,20]. The case reports on nasal and paranasal EMPs in the Turkish literature are listed in Table 2. However, follow-up information was missing for most of the patients and therefore we were unable to perform a documented analysis on disease recurrence or progression. This was the shortcoming of this report.

In summary, solitary EMP usually develops in the head and neck soft tissues but may also involve sites such as the thyroid and brain and may be observed in a large age range (17-80 years). One should always include the possibility of plasma cell tumor in older patients presenting with symptoms of epistaxis, nasal blockage, or facial mass. Deep submucosal biopsies are usually required so as to not miss the lesion in frozen sectioning. This neoplasm is usually treated with radiotherapy or with a combination of surgery and radiotherapy. Progression to MM may occur 3 to 8 years after treatment. Long-term follow-up in terms of disease recurrence and progression is mandatory.

## CONFLICT OF INTEREST STATEMENT

The authors of this paper have no conflicts of interest, including specific financial interests, relationships, and/ or affiliations relevant to the subject matter or materials included.

## Figures and Tables

**Table 1:**
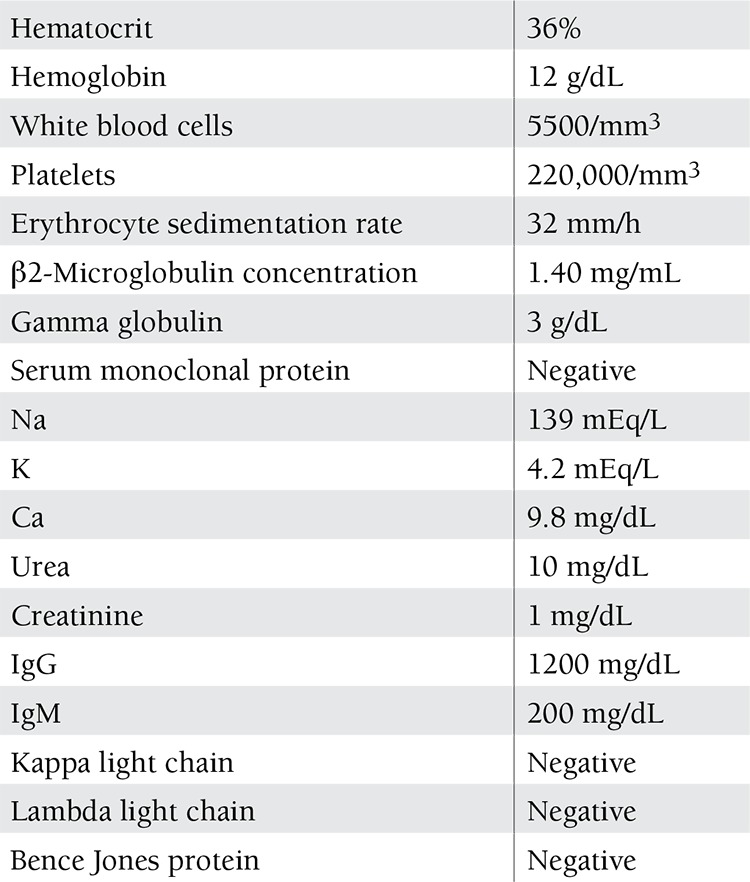
The blood work and urine electrophoresis of the patient.

**Table 2:**
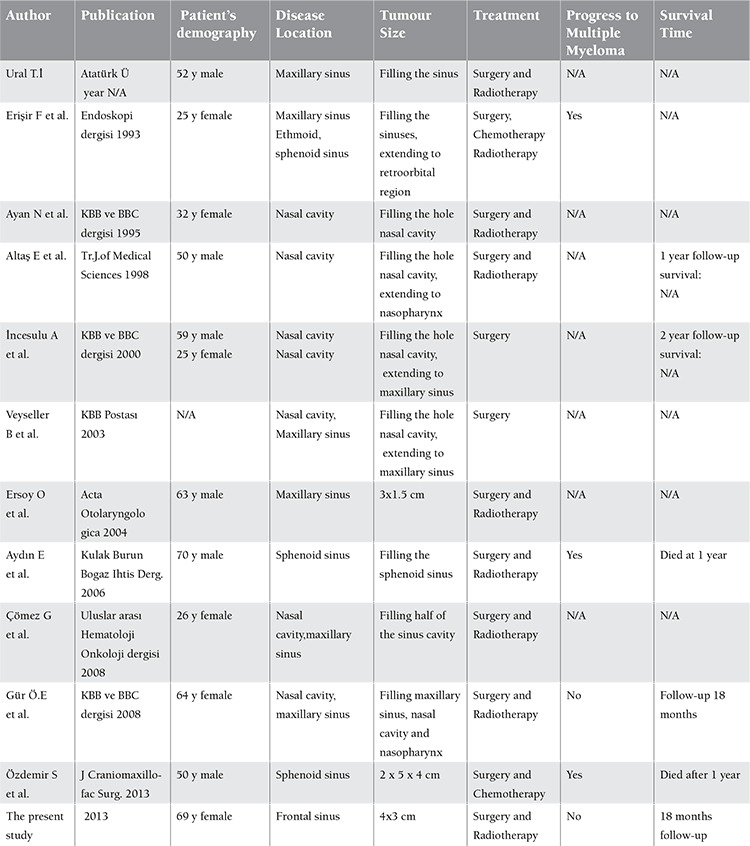
Information about nasal and sinonasal extramedullary plasmacytomas published in Turkish literature.
